# Gut Microbiome and Cytokine Profiles in Post-COVID Syndrome

**DOI:** 10.3390/v16050722

**Published:** 2024-05-02

**Authors:** Karakoz Mussabay, Samat Kozhakhmetov, Marat Dusmagambetov, Aitolkyn Mynzhanova, Madiyar Nurgaziyev, Zharkyn Jarmukhanov, Elizaveta Vinogradova, Aigul Dusmagambetova, Aiganym Daulbaeva, Laura Chulenbayeva, Ainur Tauekelova, Makhabbat Bekbossynova, Almagul Kushugulova

**Affiliations:** 1 Department of Microbiology and Virology Named after Sh.I.Sarbasova, Astana Medical University, Astana 010000, Kazakhstan; dusmagambetov.m@amu.kz (M.D.); dusmagambetova.a@amu.kz (A.D.); 2 Laboratory of Microbiome, Center for Life Sciences, National Laboratory Astana, Nazarbayev University, Astana 010000, Kazakhstan; skozhakhmetov@nu.edu.kz (S.K.); madiyar.nurgaziyev@nu.edu.kz (M.N.); zharkyn.jarmukhanov@nu.edu.kz (Z.J.); st.paulmississippi@gmail.com (E.V.); laura.chulenbayeva@nu.edu.kz (L.C.); 3 Department of Pediatric Infectious Diseases, Astana Medical University, Astana 010000, Kazakhstan; mynzhanova.a@amu.kz (A.M.); daulbaeva.a@amu.kz (A.D.); 4 National Research Cardiac Surgery Center, Astana 010000, Kazakhstan; tauekelovaajnura@gmail.com (A.T.); cardiacsurgeryres@gmail.com (M.B.)

**Keywords:** SARS-CoV-2, Post-COVID, gut microbiome, cytokines, cytokine storm

## Abstract

Recent studies highlight the crucial role of the gut microbiome in post-infectious complications, especially in patients recovering from severe COVID-19. Our research aimed to explore the connection between gut microbiome changes and the cytokine profile of patients with post-COVID syndrome. Using 16S rRNA amplicon sequencing, we analyzed the composition of the gut microbiome in 60 COVID-19 patients over the course of one year. We also measured the levels of serum cytokines and chemokines using the Milliplex system. Our results showed that severe SARS-CoV-2 infection cases, especially those complicated by pneumonia, induce a pro-inflammatory microbial milieu with heightened presence of *Bacteroides*, *Faecalibacterium*, and *Prevotella_9*. Furthermore, we found that post-COVID syndrome is characterized by a cross-correlation of various cytokines and chemokines MDC, IL-1b, Fractalkine, TNFa, FGF-2, EGF, IL-1RA, IFN-a2, IL-10, sCD40L, IL-8, Eotaxin, IL-12p40, and MIP-1b as well as a shift in the gut microbiome towards a pro-inflammatory profile. At the functional level, our analysis revealed associations with post-COVID-19 in homolactic fermentation, pentose phosphate, NAD salvage, and flavin biosynthesis. These findings highlight the intricate interplay between the gut microbiota, their metabolites, and systemic cytokines in shaping post-COVID symptoms. Unraveling the gut microbiome’s role in post-infectious complications opens avenues for new treatments for those patients with prolonged symptoms.

## 1. Introduction

Post-COVID syndrome encompasses a spectrum of physical and psychological symptoms emerging in individuals after COVID-19 recovery, while the cytokine storm in COVID-19 involves the rapid and excessive release of antiviral cytokines and inflammatory mediators due to SARS-CoV-2 infection, triggering immune hyperactivation and tissue hypoxia. Particularly, IL-1β, IL-1RA, IL-6, IL-8, IL-18, and TNF-α are associated with cytokine storm.

Hirayama et al. have found that secondary bile acids produced by gut bacteria, such as *Collinsella*, suppress cytokine storm syndrome by inhibiting pro-inflammatory cytokines [1]. Other publications showed that in response to changes towards opportunistic flora, the microbiome activates the innate immune system, induces pro-inflammatory cytokines, and contributes to cytokine storms [2,3]. Furthermore, gut microbiota plays a crucial role in the development and functionality of the host immune system and may influence COVID-19 severity [4], especially in patients with gastrointestinal symptoms, who are at higher risk of developing severe complications such as liver injury and acute respiratory distress syndrome (ARDS), leading to worse clinical outcomes [5]. Even after the acute phase of the illness has resolved, patients may experience ongoing inflammation and tissue damage, leading to long-term complications. The pathophysiological mechanisms of post-COVID complications can be significantly influenced by the gut microbiome. Dysbiotic changes in the post-COVID period were characterized by a decrease in the number of anti-inflammatory bacteria such as *Bifidobacterium* and *Faecalibacterium* and an enrichment of microbiota associated with inflammation, including *Streptococcus* and *Actinomyces* [6,7]. A decrease in the relative abundance of *Christensenellaceae*, *Ruminococcus*, *Akkermansia*, and *Bacteroides thetaiotaomicron* and an increase in the proportion of the genera *Faecalibacterium*, *Veillonella*, *Lachnospiraceae*, the *Bacteroides fragilis* group, *Proteus*, *Enterococcus*, *Enterobacter*, *and Citrobacter* was shown in patients during the post-COVID period [8,9,10]. The characteristics of the microbiota differ among populations; the results showed that a high concentration of *Collinsella* was found in the intestines of (0.29–1.1%) of study participants in Australia [11] (0.73%), Japan [1] (0.831%) and South Korea [12] (0.7%), while in the United States [13] (1.1%), Germany [14], Britain [15] (0.29%), and Italy [16] (0.60%), where high mortality rates are reported, a higher concentration was detected only in 4–18% of cases. In Brazilian patients with post-COVID syndrome were found an increase in several genera, including *Desulfovibrio*, *Haemophilus*, *Dialister*, and *Prevotella*, in addition to a decrease in beneficial microbes such as *Bifidobacterium* and *Akkermansia* [17]. A large number of studies in the field of COVID-19 and post-COVID syndromes represent contributions to the study of the constituent links of the pathogenetic mechanism in the pathogenesis of SARS-CoV-2 infection and its complications. This study focuses on the relationship between changes in the gut microbiome and cytokine profile in the post-COVID period. It is important to explore if there are lasting changes in the gut microbiome after recovering from COVID-19 and whether these changes could affect the severity of post-COVID-19 symptoms.

## 2. Methods

### 2.1. Study Design

**Figure 1:**
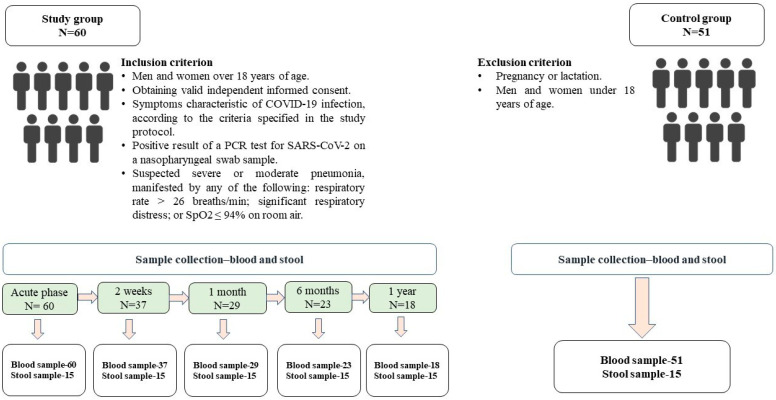
Study design.

We conducted a prospective cohort study to study the long-term consequences of COVID-19 in adult patients admitted to a modular infectious disease hospital in Astana from 5 May to 9 June 2021. The study examined the clinical course and outcome of all extremely severe cases of patients in the intensive care unit (ICU), according to the inclusion criteria, which were, in addition to symptoms and severity, mandatory PCR for COVID-19 infection, confirmed COVID pneumonia, and signed informed consent from the patient and his relatives. Relatives of critically ill patients were invited to participate in the study and provided consent on behalf of the patients, given their serious condition. A total of 60 severe patients were included in the study. Data on demographics, medical parameters, and antibiotic use during hospitalization were extracted from electronic medical records, as well as directly from patients after their condition improved and they were transferred to the internal medicine department; these data are presented in Supplementary Table S1. The design of the study is shown by the scheme in Figure 1.

### 2.2. Sample Collection

A total of 75 stool samples and 167 blood samples were collected from COVID-19 patients by professional healthcare workers. Stool samples were collected in ZymoResearch DNA/RNA shield fecal collection tubes (Cat.No:R1101) and were stored at −80 °C immediately until processing.

### 2.3. Laboratory Measurement

Determination of the blood biochemical analysis was outsourced to the commercial clinical-diagnostic laboratory “Olymp”, which routinely performs biochemical analyses. Cytokines/chemokines were analyzed in blood serum using the Milliplex HCYTMAG60PMX41BK kit (Millipore, Burlington, MA, USA) according to the manufacturer’s recommendations on a BioRad Bio-Plex 200 system (Bio-Rad Laboratories, Hercules, CA, USA). 

### 2.4. Microbiome Analysis

The DNA/RNA shield fecal collection tubes were designed with a total volume of 10 mL, comprising 9 mL of lysis buffer and 1 mL of fecal material each. The DNA extraction utilized the ZymoBiomics DNA Microprep kit (Cat. No: D4300) (Zymo Research Corporation, Tustin, CA, USA). A standardized 1 mL of material was extracted from each DNA/RNA shield fecal collection tube. DNA concentrations were measured using a Nanodrop2000/2000c (ThermoFisher, Waltham, MA, USA). DNA sequencing was performed on the Illumina NovaSeq6000 (Illumina, Inc., San Diego, CA, USA). PICRUSt was used to predict functional content from the 16S rRNA amplicons. Data analysis was performed using less operational taxonomic units scripts 2 (LotuS2).

### 2.5. Statistical Analysis

The distribution of key study population characteristics by COVID-19 status was analyzed using the Mann–Whitney U-test for continuous variables and the χ2 test for categorical variables. A *p*-value of <0.05 was considered statistically significant.

Within-sample community diversity (α-diversity) was assessed using the Shannon and Simpson indices and the observed and estimated number of taxa (observed and Chao1 index, respectively) and compared between groups using a Mann–Whitney U-test for two independent groups (control vs. T1) or Friedman test for dependent groups with the Wilcoxon post hoc test (with FDR, BH correction, *p*-value < 0.05) for dependent groups (T1–T5). Only those OTUs that were present in at least 25% of the samples were included. Divergence in community composition between samples (β-diversity) was assessed by calculating the Bray–Curtis metric (abundance). Before calculating the β-diversity, data were transformed using the Hellinger transformation. Ordination was visualized using principal coordinate analysis (PCoA) and compared using ANOSIM tests with 999 permutations. Diversity calculation, ordination, ANOSIM, and PERMANOVA tests were performed in Python 3 using the “scikit-bio0.5.6” package. STAMP 2.1.3 software was used to identify significant functional differences between experimental groups. LEfSe was used to identify the most differentially distributed taxa. Metabolic pathways, as well as OTUs, were derived from the MetaCyc microbial database. Associative networks were constructed using the Spearman correlation coefficient for continuous variables and Point-Biserial for mixed binary–continuous pairs. Association was considered significant at (FDR, BH, *p*-value < 0.05). All other statistical calculations were performed in Python 3 using “SciPy 1.7.0,” and visualization was performed using the “matplotlib 3.7.0” library.

## 3. Results

### 3.1. Characteristics of Gut Microbiome and Cytokines of COVID-19 and Non-COVID-19 Controls

**Figure 2:**
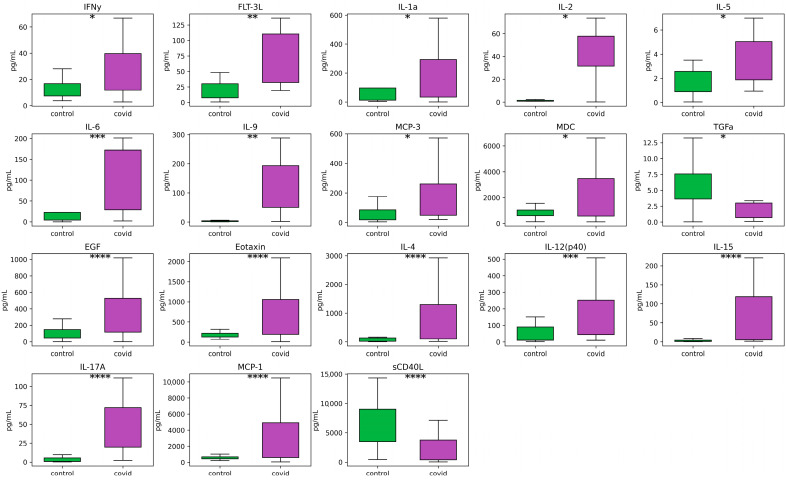
Box plot representations of cytokine expression levels of COVID-19 (*n* = 60) and control (*n* = 51) groups. The x-axis shows healthy and COVID-19 severe groups, and the y-axis is analyte concentration in pg/mL. Mann–Whitney U-test, with an FDR corrected * *p*-value ≤ 0.05, ** *p*-value ≤ 0.001, *** *p*-value ≤ 0.0001, **** *p*-value ≤ 0.00001.

Our findings indicate a significant elevation of various cytokines, both pro-inflammatory and anti-inflammatory, in the COVID-19 group (acute phase of disease) compared to the control. Notably, pro-inflammatory cytokines such as IL-1a (FDR = 0.03047), FLT-3L (FDR = 0.00302), IL-2 (FDR = 0.03924), IL-6 (FRD = 0.00048), IL-9 (FDR = 0.00778), MCP-3 (FDR = 0.01850), IFNy (FDR = 0.06763), and IL-17A (FDR = 0.00012) exhibited significant increases. Additionally, anti-inflammatory cytokines including IL-5 (FDR = 0.09064), EGF (FDR = 0.00012), Eotaxin (FDR = 0.00012), IL-4 (FDR = 0.00001), IL-12(p40) (FDR = 0.00082), MDC (FDR = 0.05648), MCP-1 (FDR = 0.00012), and IL-15 (FDR = 0.00004) were also found to be elevated (Figure 2).

**Figure 3:**
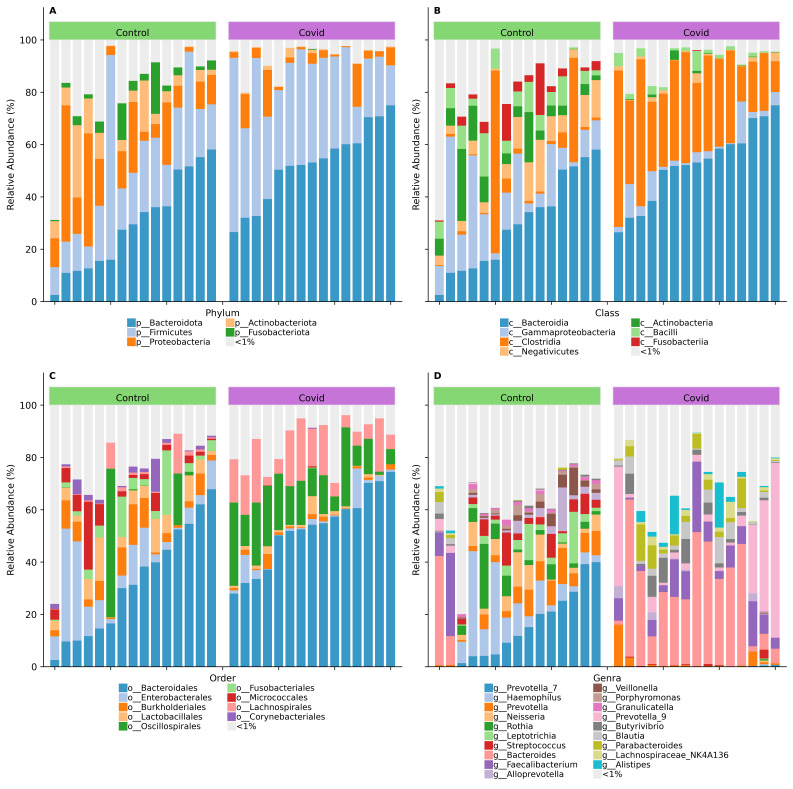
Stacked barplots (**A**) Phylum, (**B**) Class, (**C**) Order, (**D**) Genus showing the relative abundance of bacterial OTUs in control (*n* = 15, **left**) and COVID-19 (*n* = 15, **right**) samples, classified into levels. Relative abundance is represented by the height of the bar. Only taxa with the highest relative abundance (>1%) at each level are displayed.

Compositional analysis of gut microbiome of COVID-19 (acute phase of disease) showed dominance of *Bacteroidota* and *Firmicutes* (Figure 3A, Supplementary Table S2) and a reduction in *Actinobacteria*, *Fusobacteria*, and *Proteobacteria*; at the class level, a decrease in *Actinobacteria*, *Gammaproteobacteria*, and *Negativicutes* and an increased abundance of *Bacteroidia* and *Clostridia* (Figure 3B); at the order level, a significant decrease in *Oscillospirales* and *Micrococcales* and increased *Bacteroidales* and *Lachnospirales* (Figure 3C). At the genus level, the study group exhibited a higher abundance of *Bacteroides*, *Feacalibacterium*, and *Prevotella_9* and, conversely, there was a reduction in *Haemophilus*, *Leptotrichia*, *Prevotella*, *Prevotella_7*, *Neisseria*, and *Streptococcus*. 

**Figure 4:**
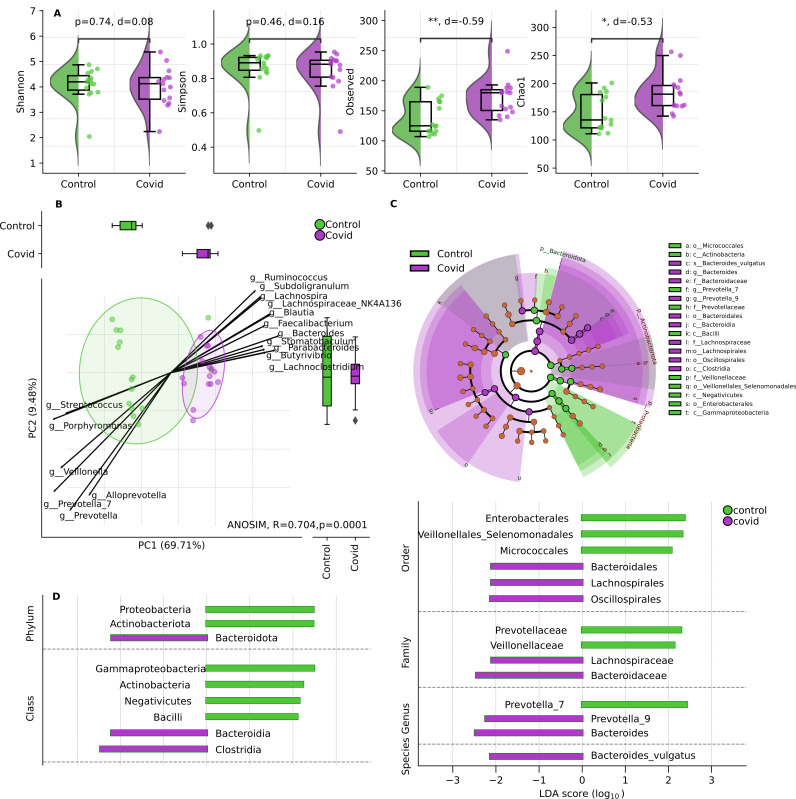
(**A**). Alpha biodiversity between control (*n* = 15) and COVID-19 (*n* = 15) groups utilizing Shannon, Simpson, observed, and Chao1 indices. Mann–Whitney U-test, * *p*-value ≤ 0.05, ** *p*-value ≤ 0.001, and d-value indicating effect size. (**B**). Beta diversity. PCoA ordination of community membership based on Bray–Curtis distance in control and COVID-19 groups with ANOSIM test for degree of separation. (**C**,**D**). Linear discriminant analysis (LDA) with effect size (LEfSe). (**C**). Cladogram showing the phylogenetic distribution of the microbiota discriminating between the control and COVID-19 groups. The central point marks the root of the tree and extends to lower taxonomic levels from phylum to species. The diameter of each circle is proportional to the abundance of the taxon. (**D**). Key discriminating phylotypes of taxa, LDA score > 2.0. Taxa enriched in the control group are indicated by a positive LDA score (green). Taxa enriched in the COVID-19 group are indicated by a negative score (purple).

Based on the alpha diversity analysis using the Shannon and Simpson indices, the study did not find a statistically significant difference; the observed richness and Chao1 showed a statistically significant difference (Figure 4A). Beta diversity analysis using Bray–Curtis distance with analysis of similarities (ANOSIM) was performed to compare the microbial community structure and indicated a significant difference in the microbial community structure between the COVID-19 (acute phase of disease) and control groups. (Figure 4B). The compositional differences of abundance at different taxonomic levels were confirmed by LEfSe (Figure 4C,D, Supplementary Table S3). 

Furthermore, functional biomarkers of bacterial genes are investigated in Supplementary Figure S1 (Supplementary Table S4). The dominance of the carbohydrate metabolism module in the COVID-19 group was revealed, with the exception of the (R,R)-butanediol biosynthesis superpathway and glucose and glucose-1-phosphate degradation, which were more abundant in healthy controls. It is consistent with the findings [18] that dysregulation of glucose metabolism leads to an increase in opportunistic bacteria. Metabolism of cofactors and prosthetic groups, electron carriers, and nucleosides/nucleotides exhibited decreased activity across all pathways in the COVID-19 group, except for NAD biosynthesis I, which showed a slight increase. Amino acid and vitamin metabolism displayed a consistent increase, while fatty acid and lipid metabolism showed decreased activity.

There is evidence of an association of the genus *Gemella* with respiratory tract infections [19] and our analysis demonstrated a positive correlation in Supplementary Figure S2 with closely related IL4 and IL-13, which are Th2 cytokines, and associated with a type-2 immune response [20,21]. In turn, IL-13 and IL-4 show independent positive correlations with IFN-a2 and FGF-2, which may be a compensatory response to Th2-driven inflammation. Dysregulation of glycogen biosynthesis, NAD salvage, and L-alanine biosynthesis pathways, which are involved in cellular metabolism, can exacerbate infectious processes. IFN-γ has a significant positive correlation with the *Prevotella* genus and negative correlation with the *Bacteroidaceae* family.

Furthermore, MIP-1b is positively correlated with IP-10, IL-10, and G-CSF in COVID-19 patients. IP-10 has been shown to be elevated in COVID-19 patients and may play a role in attracting T-cells to the lungs. IL-10 has been hypothesized to play a role in regulating the immune response in COVID-19 patients, while G-CSF has been shown to stimulate the production of neutrophils, which contributes to lung inflammation.

IL-1A has been found to be positively correlated with the degradation of adenosine nucleotides II, guanosine nucleotides III, and purine nucleotides II, while negatively correlated with 4-deoxy-L-threo-hex-4-enopyranuronate degradation. Also, in Supplementary Figure S2B, we observe that IL-1A was positively correlated with pathways involved in the degradation of purine and guanosine nucleotides, as well as adenosine-nucleotide degradation-II, while being negatively with the 4-deoxy-L-threo-hex-4-enopyranuronate degradation pathway. IL-1A may contribute to the dysregulation of nucleotide metabolism and may lead to the accumulation of nucleotides and some metabolites that may have a pro-inflammatory effect and contribute to the pathogenesis of the disease [22]. Nucleotides are important building blocks of DNA and RNA and are involved in many cellular processes. However, excessive accumulation of nucleotides can be toxic and trigger inflammatory reactions [23,24]. There is evidence of increased nucleotide biosynthesis and decreased nucleotide catabolism in COVID-19, which can lead to accumulation of nucleotides and their metabolites. This accumulation can activate immune cells and trigger pro-inflammatory responses, contributing to the pathogenesis of the disease.

In addition, some studies have shown that the accumulation of nucleotides and their metabolites can also contribute to the cytokine storm observed in severe cases of COVID-19 [25].

Fractalkine has been found to positively correlate with Eotaxin and IL-1A in patients with COVID-19. Eotaxin has been shown to be elevated in patients with COVID-19 and it has been suggested that it may play a role in recruiting eosinophils, which may contribute to lung injury [26].

### 3.2. Gut Microbiome and Cytokine Profile in Dynamics

A significant difference in gut composition over time was observed in the COVID-19 group, as indicated by changes in species richness in Supplementary Figure S3. The effect sizes varied depending on the time points. The largest differences were observed between T4/T5 and earlier time points with large to very large effect sizes. Differences between T1/T2 and T3/T4 were generally smaller, with small to moderate effect sizes.

**Figure 5:**
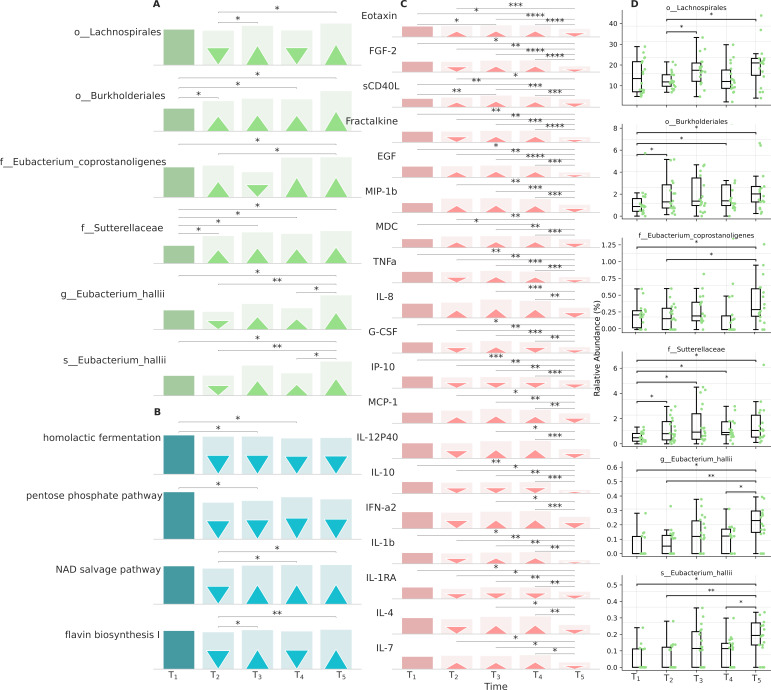
An overview of gut composition, metabolic pathways, and immune features that showed significant changes in the COVID-19 group over time. (**A**–**C**). The abundance of taxonomic features (green), metabolic pathways (blue), and immune markers (red). Marker size indicates the average abundance of each component and the direction of change (upwards-facing: enriched, downwards-facing: depleted) compared to baseline (square markers, T0). (**D**). Relative abundance of taxonomic features. Friedman, Wilcoxon, FDR, * *p*-value ≤0.05, ** *p*-value ≤0.001, *** *p*-value ≤ 0.0001, **** *p*-value ≤ 0.00001.

The dynamics of the compositional gut microbiome at T1–T5 points at the level of phylum, class, order, and genus is shown in Supplementary Figure S4. Additionally, Supplementary Table S5 provides a detailed representation of the data, including error bars for enhanced clarity in the interpretation of the results. Across the initial 6-month period from T1 to T4, the gastrointestinal tract exhibited a predominance of microbial classes, notably *Bacteroidia* and *Clostridia*, accompanied by a decreased presence of *Negativicutes*, *Alphaproteobacteria*, and *Gammaproteobacteria*. Subsequent to the T4 time point, indicative of one year, a noticeable decline in opportunistic flora was discerned, coinciding with a statistically significant rise in commensal microbial populations. Moreover, several statistically significant taxa of bacteria are identified, including at the order level *Coriobacteriales*, *Erysipelotrichales*, *Lachnospirales*, and *Burkholderiales*, at the family level *Tannerellaceae*, the *[Eubacterium] coprostanoligenes* group, and *Sutterellaceae*, and at the genus level *Parabacteroides* and the *E. hallii* group (Figure 5). 

Further, using PICRUSt2, we predicted major metabolic pathways and found depletions in homolactic fermentation and the pentose phosphate pathway throughout the observation period. NAD-salvage pathway-I and flavin biosynthesis-I were depleted during the acute T1–T2 stage and enriched a month later at T3–T5. Chitin derivative degradation was initially enriched, then depleted at T3–T5, while aerobactin biosynthesis was gradually depleted throughout the observation. The depletion and subsequent enrichment of NAD-salvage pathway-I and flavin biosynthesis-I may indicate a shift in gut microbiota metabolic needs during COVID-19 infection.

**Figure 6:**
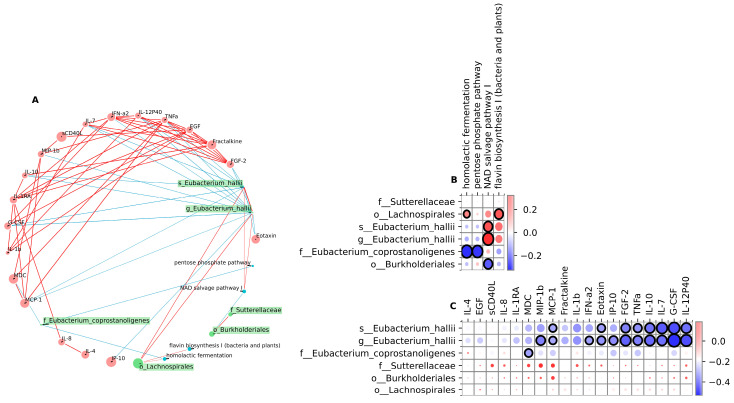
Correlation analysis. (**A**) Network plot showing the co-occurrence between cytokines and chemokines (red nodes), taxa (green nodes), and pathways (blue nodes) in the COVID-19 group over time. Node size represents average feature abundance. (**B**) Heatmap correlation between taxa of bacteria and metabolic pathways. (**C**) Heatmap correlation between taxa of bacteria and cytokines and chemokines. Positive correlations are represented in red and negative correlations in light blue. Spearman, FDR, *p* < 0.05.

It is found that *Eubacterium coprostanoligenes* exhibited negative correlations with homolactic fermentation and pentose phosphate pathways (Figure 6), which are known to play a crucial role in energy metabolism and immune response [27]. Positive correlations between NAD-salvage pathway-I and *Eubacterium halii* are observed, and negative with *Burkholderiales*. Additionally, we found a positive correlation between *Lachnospirales* and the flavin biosynthesis-I pathway. 

Finally, we identified the cytokines that exhibit significant changes in dynamics in the post-COVID period (Figure 5A T4-N5, Supplementary Table S6). A reduction in immune markers at the end of the one-year study, as compared to the six-month period, was determined. However, the levels of cytokines such as Eotaxin (*p* = 0.01619), MCP-1 (*p* = 0.00794), MDC (*p* = 0.00169), MIP-1a (*p* = 0.00072), TGFa (*p* = 0.00111), TNFa (*p* = 0.06176), and VEGF-A (*p* = 0.02385) decreased, while FGF-2 (*p* = 0.01133), G-CSF (*p*= 0.01133), and IL-15 (*p* = 0.03340) were increased post-COVID. 

Our results showed cross-correlation of MDC, IL-1b, Fractalkine, TNFa, FGF-2, EGF, IL-1RA, IFN-a2, IL-10, sCD40L, IL-8, Eotaxin, IL-12p40, and MIP-1b; this confirms the previously published data and their associations with the severity of coronavirus infection [28]. A similar cross-correlation was found for IL-7, G-CSF, MIP-1b, and MCP-1 in dynamics, which is also associated with the severity of SARS-CoV-2 infection [29]. 

A significant negative correlation of MCP-1, MIP-1b, IFN-a2, eotaxin, TNFa, IL-7, IL-12P40, FGF-2, IP-10, and IL-10 with *Eubacterium halii* at the genus and species levels has been established (Figure 6). It should be noted that IL-6 critically increases in the acute period and remains elevated throughout the entire observation period without significant changes compared to the T0 point, and IL-1RA even by the 6th month retains a downward trend, while IL-1a is elevated, indicating an ongoing inflammatory process. 

## 4. Discussion

The intestinal microbiome with produced metabolites, in combination with systemic immunity, makes a significant contribution to the complex multi-level pathogenetic mechanism for the development of post-COVID syndrome. In this study, we evaluate the fecal microbiota of Kazakhstan patients in different post-COVID periods and correlate this with serum cytokines patterns. 

Severe SARS-CoV-2 infection complicated by pneumonia is characterized by a shift towards the pro-inflammatory phenotype with an increase in IL-1a, FLT-3L, IL-2, IL-6, IL-9, MCP-3, IFNy, IL-5, Eotaxin, IL-12(p40), MDC, IL-17A, MCP-1, and IL-15, and on the part of the fecal microbiome, with dominance of *Bacteroides*, *Feacalibacterium*, and *Prevotella_9* and a reduction in *Haemophilus*, *Leptotrichia*, *Prevotella*, *Prevotella_7*, *Neisseria*, and *Streptococcus*. In a study involving 294 COVID-19 patients, the analysis revealed key post-COVID cytokines—FGF-2, VEGF-A, EGF, IL-12(p70), IL-13, and IL-6—crucial for understanding pathophysiology. Complications like arterial hypertension, diabetes, and others may arise within six months post-recovery, irrespective of disease severity [30]. 

In our study, post-COVID syndrome, accompanied by prolonged symptoms of headache, weakness, fatigue, and sleep disorders [9], is characterized by cross-correlation of MDC, IL-1b, Fractalkine, TNFa, FGF-2, EGF, IL-1RA, IFN-a2, IL-10, sCD40L, IL-8, Eotaxin, IL-12p40, and MIP-1b and a shift in the gut microbiome towards a pro-inflammatory microflora. Zhou et al. identified several opportunistic pathogens enriched in recovered COVID-19 patients after 3 months, including *Escherichia unclassified*, *Intestinibacter bartlettii*, *Clostridium aldenense*, *Clostridium bolteae*, *Flavonifractor plautii*, and *Clostridium ramosum*, which are associated with COVID-19 severity compared to healthy controls (HCs) [31]. These results mirror our findings after a 6-month period. During this time frame (T1 to T4), there was a predominance of the microbial classes *Bacteroidia* and *Clostridia* in the gastrointestinal tract, accompanied by a reduced presence of *Negativicutes*, *Alphaproteobacteria*, and *Gammaproteobacteria.* Zhang and colleagues reported notable variations in the relative abundance of the bacterial genera *Eubacterium*, *Agathobacter*, *Subdoligranulum*, and *Ruminococcus* among COVID-19 patients who developed long COVID, with *Veillonella* being notably overrepresented in long COVID patients one year after infection [32]. Interestingly, our study did not find an enrichment of *Veillonella* in the COVID-19 group, but we observed similar results with other genera. Nevertheless, we observed considerable long-term clinical consequences of coronavirus infections, the severity of which correlated with the composition of the intestinal microbiota. Mare’s milk has been shown to counteract the shift towards a pro-inflammatory gut microbiome, fostering the recovery of gut health and alleviating prolonged symptoms [33]. 

Most of the results are in agreement with those previously published [17,34,35,36]. At the functional level, the associations with post-COVID in homolactic fermentation, pentose phosphate, NAD-salvage, and flavin biosynthesis-related genes are revealed. 

The homolactic fermentation and pentose-phosphate pathways can contribute energy metabolism and SCFA pro1duction. Alterations in the activity of the pentose phosphate pathways, or disbalance of bacteria that used this pathway, like *Bacteroides* and *Prevotella* in our study, can lead to conditions such as obesity, type 2 diabetes, and inflammatory bowel disease [37]. Under conditions of high energy demand and oxidative stress, as in our study with coronavirus infection, NAD+ uptake may exceed its synthesis with subsequent depletion. In turn, it leads to a violation of cellular functions, subsequently manifested by metabolic disorders and cognitive dysfunction [38]. In addition, NAD+ depletion is associated with increased production of pro-inflammatory cytokines such as IL-1beta, IL-6, and TNF-alpha [39], while in our study it negatively correlates with MCP-1 and IP-10. Flavins also affect immune function; some studies suggest that depletion of flavin biosynthesis may be associated with changes in cytokine levels and immune function [40].

The metabolic pathways mentioned are linked to beneficial bacteria such as *Lactobacillus* and *Bifidobacterium*, which were significantly reduced in the study group and in post-COVID dynamics.

Our study suggests a relationship between a range of post-COVID symptoms and complex interactions between gut microbiota with their metabolites and systemic cytokines. Reduced *E. hallii* is a member of the beneficial intestinal flora and maintains the balance of intestinal metabolism due to its ability to utilize glucose and acetate and lactate fermentation intermediates to form butyrate and hydrogen, unlike other intestinal bacteria that produce butyrate from monosaccharides [41]. In our study, *E. hallii* was significantly reduced during the acute period, but a month after the onset of the disease, a persistent upward trend was observed. Limited research on the role of *E. hallii* specifically in COVID-19 has been conducted, but studies suggest that alterations in the gut microbiota, including changes in the abundance of *E. hallii*, may be associated with COVID-19 severity [42]. Studies have found that COVID-19 patients had a significant decrease in the abundance of *E. hallii* and other beneficial gut bacteria compared to healthy controls, and the depletion of these bacteria was associated with markers of inflammation and disease severity in COVID-19 patients [1]. In our study, we found in the dynamics of the study that *E. hallii* negatively correlates with MIP-1b, MCP-1, IFNa2, Eotaxin, TNFa, IL-7, IL-12(p40), G-CSF, IP-10, IL-10, and FGF-2. It is suggested that *E. hallii* may impact the immune system and cytokine production; it is associated with the production of anti-inflammatory cytokines and decreased levels of pro-inflammatory cytokines in human gut epithelial cells [43]. *E. hallii* may be a marker of both insulin resistance and cognitive dysfunction; however, this requires more research.

This study has several limitations. The relatively small sample size (*n* = 15 for gut metagenome and *n* = 60 for immune cytokine profiles) may make it difficult to establish a specific association, despite our efforts to apply a rigorous correction for false positives. However, our findings appear to be supported and strengthened by the existing research, which we hope to expand upon by providing new data. Second, our study included patients with extremely severe disease compared to healthy controls, but not in a comparison with mild, moderate, and severe disease. For this reason, it is not possible to analyze markers by severity. However, this was not our goal, but to study how the parameters of the stool microbiome and serum cytokines change during a year of severe COVID-19 infection complicated by pneumonia. Third, we do not have information on the circulating SARS-CoV-2 strains present in the study population. However, given that patient recruitment occurred over a narrow time period, it is possible to assume the possibility of infection with the same strain.

## 5. Conclusions

Post-COVID syndrome demonstrates the significant involvement of the gut microbiome and systemic cytokines in its development. A shift towards a pro-inflammatory gut microflora is observed in severe COVID-19 cases, especially those complicated by pneumonia. Prolonged symptoms in post-COVID patients are associated with specific cytokine patterns and altered microbial profiles, highlighting a complex interplay between microbiota, metabolites, and immune responses.

## Data Availability

The datasets presented in this study can be found in online repositories. The names of the repository/repositories and accession number(s) can be found below: Temporary SubmissionID: SUB13279337. Release date: 20 June 2024.

## Supplementary Materials

The following supporting information can be downloaded at: https://www.mdpi.com/article/10.3390/v16050722/s1, Figure S1: Gut microbiome metabolic pathways; Figure S2: Correlation analysis; Figure S3. Alpha diversity in the covid-19 group over time; Figure S4. Stacked barplots showing the average relative abundance of bacterial OTUs in the acute period; Table S1: Demographics, baseline characteristics and laboratory characteristics of control and COVID-19 patients according to gender; Table S2: Relative Abundance of Bacterial Taxa in control and covid group; Table S3: Linear discriminant analysis (LDA) results with effect size in control and covid group; Table S4: Metabolic Pathways with Significant FDR in control and covid group; Table S5. Compositional Gut Microbiome with Error Bars in covid group over time; Table S6: Abundance of taxonomic features, metabolic pathways, and immune markers in covid group over time.
