# Supplementary material for: Gut Microbiome and Cytokine Profiles in Post-COVID Syndrome
Source: Viruses. 2024 May 2;16(5):722. doi: 10.3390/v16050722 (PMC11126011; doi:10.3390/v16050722)
Supplement: Supplementary file 1 [file viruses-16-00722-s001.zip › Figure S1.pdf]

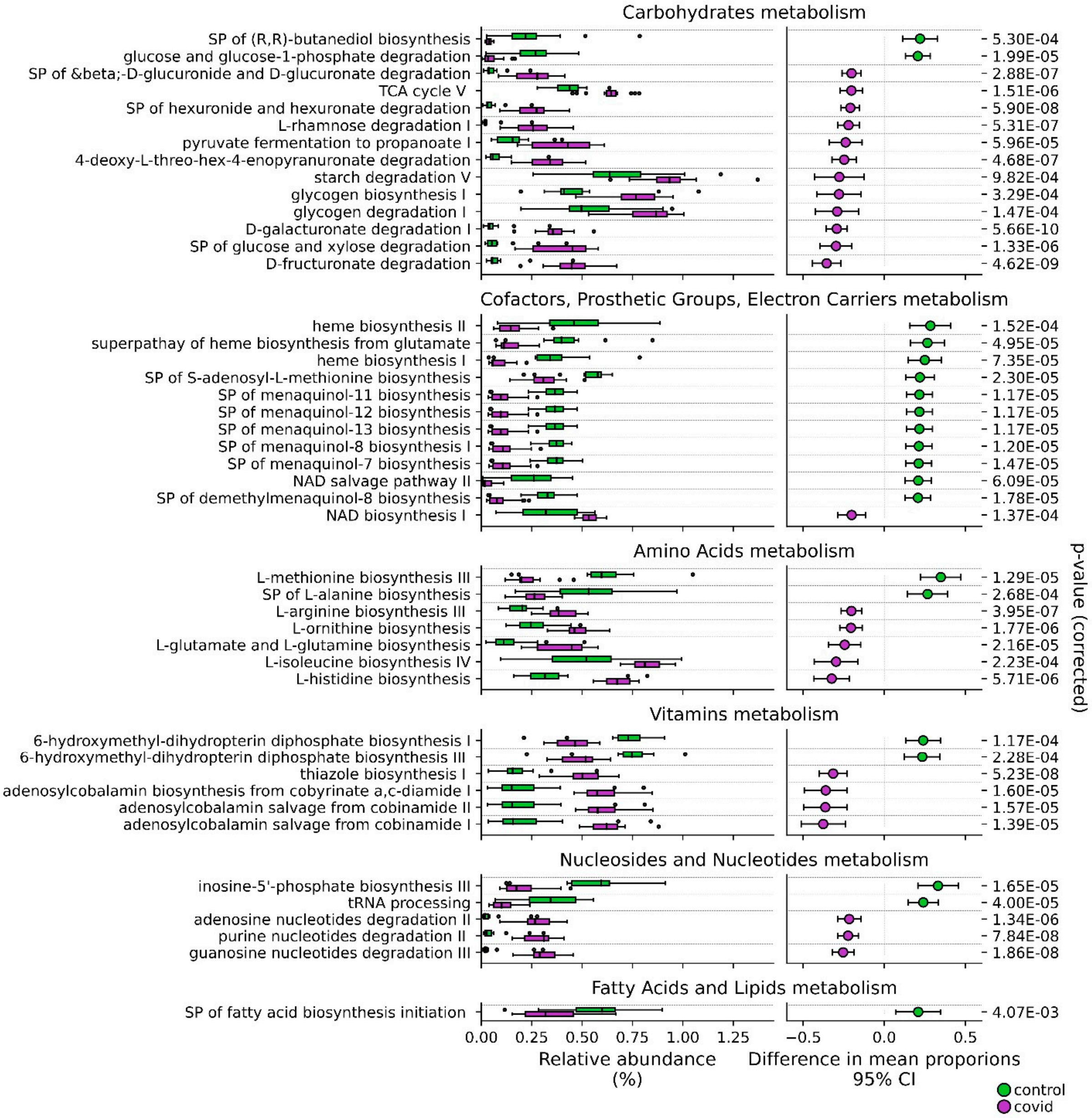

Supplementary Figure S1. Gut microbiome metabolic pathways. Extended error bar (EEB) plots showing functional properties that significantly differ (FDR, $p<0.05$) between control and covid-19 gut microbiomes. The left side shows relative abundance of metabolic features based on gut microbiome abundance, and the right side visualizes the difference in mean proportions between groups for each feature. Green indicates control group; purple indicates covid-19 group
