# Supplementary material for: Gut Microbiome and Cytokine Profiles in Post-COVID Syndrome
Source: Viruses. 2024 May 2;16(5):722. doi: 10.3390/v16050722 (PMC11126011; doi:10.3390/v16050722)
Supplement: Supplementary file 1 [file viruses-16-00722-s001.zip › Figure S2.pdf]

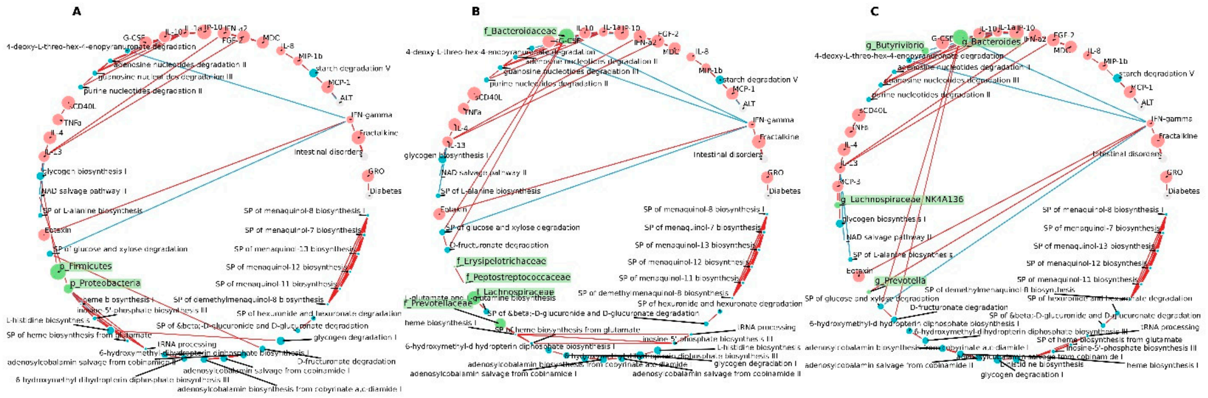

Supplementary Figure S2. Correlation analysis. Network plot showing the co-occurrence between cytokines and chemokines (red nodes), taxa (green nodes), and pathways (blue nodes) in the covid-19 group. 2A Phylum, 2B Family, 2C Genus level. Red lines represent positive correlation and blue lines represent negative correlation. Node size represents average feature abundance. Spearman, FDR, $p<0.05$
