# Supplementary material for: Gut Microbiome and Cytokine Profiles in Post-COVID Syndrome
Source: Viruses. 2024 May 2;16(5):722. doi: 10.3390/v16050722 (PMC11126011; doi:10.3390/v16050722)
Supplement: Supplementary file 1 [file viruses-16-00722-s001.zip › Figure S3.pdf]

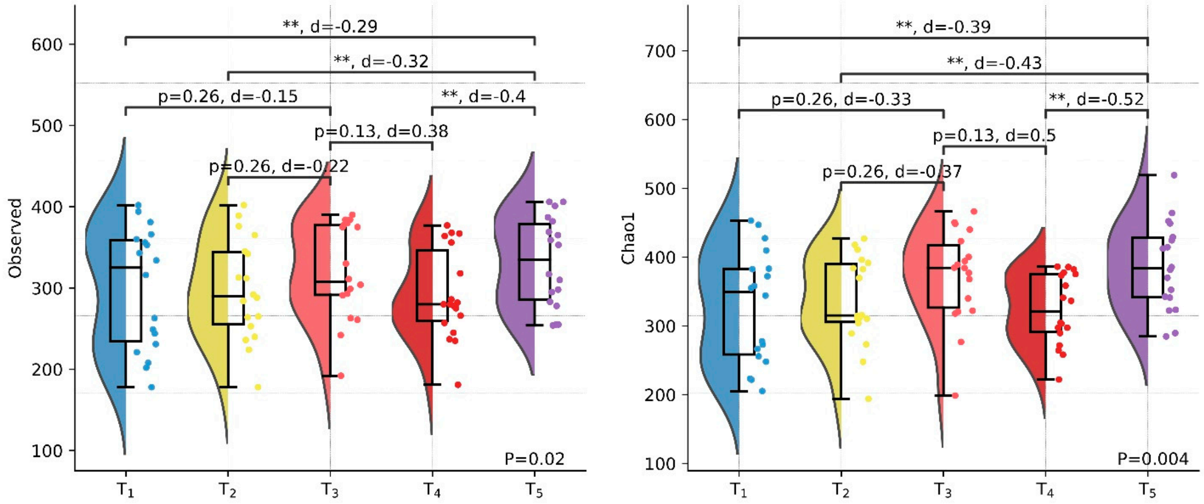

Supplementary Figure S3. Alpha diversity in the covid-19 group over time. Observed (left) and estimated (right, Chao1) number of taxonomic features in the acute period - T1, 2 weeks - T2, 1 month - T3, 6 months - T4 and 1 year - T5 after disease onset. Friedman test with post hoc Wilcoxon multiple comparison test, FDR, $p<0.05$. Cliff's delta (d) effect size indicates the magnitude of the difference
