# Supplementary material for: Gut Microbiome and Cytokine Profiles in Post-COVID Syndrome
Source: Viruses. 2024 May 2;16(5):722. doi: 10.3390/v16050722 (PMC11126011; doi:10.3390/v16050722)
Supplement: Supplementary file 1 [file viruses-16-00722-s001.zip › Figure S4.pdf]

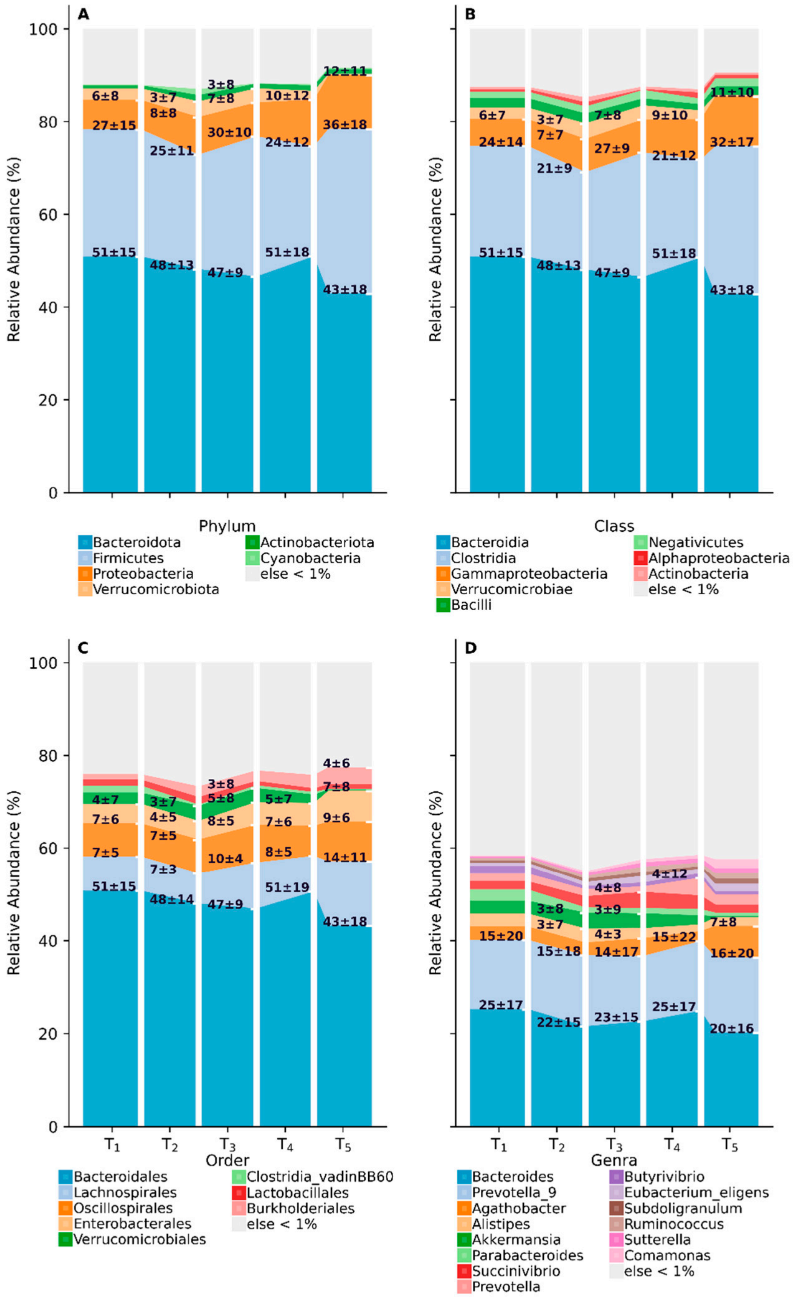

Supplementary Figure S4. Stacked barplots showing the average relative abundance of bacterial OTUs in the acute period - T1, 2 weeks - T2, 1 month - T3, 6 months - T4 and 1 year - T5 after disease onset, classified into: 5A Phylum, 5B Class, 5C Order, 5D Genus levels. Relative abundance is represented by the height of the bar. Only taxa with the highest relative abundance (>1%) at each level are displayed
