# Supplementary material for: Gut Microbiome and Cytokine Profiles in Post-COVID Syndrome
Source: Viruses. 2024 May 2;16(5):722. doi: 10.3390/v16050722 (PMC11126011; doi:10.3390/v16050722)
Supplement: Supplementary file 1 [file viruses-16-00722-s001.zip › Table S1.pdf]

Supplementary Table S1. Demographics, baseline characteristics and laboratory characteristics of control and COVID-19 patients according to gender.

Characteristic	Healthy Control		Covid Group		p - value
	Female n=26	Male n=25	Female n=30	Male n=30	
**Demographics**					
Age (median, IQR)	49(28, 65)	56 (36, 68)	63 (34, 88)	60 (26, 90)	0.56
**Comorbidities (n, %)**					
Obesity	7 (27%)	6 (24%)	22 (76%)	13 (50%)	0.05
Arterial hypertension	-	3 (12%)	19 (63%)	11 (48%)	0.06
Cardiovascular diseases	-	2 (8%)	10 (37%)	7 (32%)	0.57
Diabetes	-	-	8 (29%)	5 (21%)	0.2
Intestinal disorders	-	-	9 (32%)	13 (54%)	0.2
Pneumonia	-	-	30 (100%)	30 (100%)	
Active cancer	-	-	4 (14%)	0 (0%)	
Respiratory failure	-	-	10 (36%)	9 (37%)	
**Laboratory result**					
Hemoglobin, g/dL	131.5 (101, 162)	137.3 (93, 164)	130 (63, 166)	137 (95, 164)	0.07
Red blood cells, 10^3µL	4.3 (3.78, 5.20)	4.42 (3.78, 519)	4.68 (2.70, 7.30)	4.90 (3.24, 5.59)	0.2
Platelet, 10^3µL	233.1 (141, 321)	235.76 (87, 402)	207 (108, 452)	233 (150, 344)	0.16
White blood cell, 10^3µL	5.7 (4.4, 9.7)	6.26 (3.1, 8.7)	6.42 (2.3, 28.2)	7.04 (3.3, 14.7)	
Lymphocytes, 10^3µL	36.6 (31.5, 56.5)	34.32 (14.5,47.2)	22.9 (6, 47)	18.2 (0.5, 96)	0.19
Stab neutrophils	-	-	8.1 (1, 29)	9.05 (2, 30)	0.34
Segmented neutrophils	-	-	59.5 (1, 78)	68.1 (5, 91)	0.08
ESR, mm/h	-	-	23.1 (2, 42)	11.7 (4, 29)	0.006
Total protein mg/dL	-	-	67.06 (59, 77.23)	64.8 (56, 75.88)	0.06
Albumin, g/dL	-	-	36.6 (32, 47.45)	37.8(29.69,45.8)	0.2
Glucose, mg/dL	5.3 (4.45, 8.37)	5.4 (4.07, 8.67)	6.51 (3, 7.69)	7.04 (3.8, 15.8)	0.28
Urea mg/dL	12.1 (7.39, 18.3)	11.8(5.71, 24.63)	5.93 (3.13, 14)	6.85(3.05, 17.84)	0.21
Total bilirubin, mg/dL	10.79(5.64,18.8)	16.87(9.8, 40.36)	15.9 (11.5, 28)	18.2 (7, 38.42)	0.11
AST, U/L	20.7 (14, 41.6)	24.3 (12.5, 38.8)	39.2 (11, 82.31)	40.2 (20, 107.66)	0.4
ALT, U/L	14.5 (8.1, 50.3)	23.4 (9.3, 77.8)	30.9(11.57,75.8)	34.5(11.56,153.)	0.3
**Abbreviations:** ESR: erythrocyte sedimentation rate, AST: aspartate aminotransferase), ALT: alanine transaminase.					
